# Small Molecule Potentiator of Adjuvant Activity Enhancing Survival to Influenza Viral Challenge

**DOI:** 10.3389/fimmu.2021.701445

**Published:** 2021-09-28

**Authors:** Tetsuya Saito, Yukiya Sako, Fumi Sato-Kaneko, Tadashi Hosoya, Shiyin Yao, Fitzgerald S. Lao, Jonathan Shpigelman, Karen Messer, Minya Pu, Nikunj M. Shukla, Michael Chan, Paul J. Chu, Howard B. Cottam, Tomoko Hayashi, Dennis A. Carson, Maripat Corr

**Affiliations:** ^1^ Moores Cancer Center, University of California San Diego, La Jolla, CA, United States; ^2^ Department of Rheumatology, Graduate School of Medical and Dental Sciences, Tokyo Medical and Dental University (TMDU), Tokyo, Japan; ^3^ The Herbert Wertheim School of Public Health and Longevity, University of California San Diego, La Jolla, CA, United States; ^4^ Department of Medicine, University of California San Diego, La Jolla, CA, United States

**Keywords:** vaccine, adjuvant, influenza virus, Toll-like receptor4, monophosphoryl lipid A, NF-κB

## Abstract

As viruses continue to mutate the need for rapid high titer neutralizing antibody responses has been highlighted. To meet these emerging threats, agents that enhance vaccine adjuvant activity are needed that are safe with minimal local or systemic side effects. To respond to this demand, we sought small molecules that would sustain and improve the protective effect of a currently approved adjuvant, monophosphoryl lipid A (MPLA), a Toll-like receptor 4 (TLR4) agonist. A lead molecule from a high-throughput screen, (*N*-(4-(2,5-dimethylphenyl)thiazol-2-yl)-4-(piperidin-1-ylsulfonyl)benzamide, was identified as a hit compound that sustained NF-κB activation by a TLR4 ligand, lipopolysaccharide (LPS), after an extended incubation (16 h). *In vitro*, the resynthesized compound (**2D216**) enhanced TLR4 ligand-induced innate immune activation and antigen presenting function in primary murine bone marrow-derived dendritic cells without direct activation of T cells. *In vivo* murine vaccination studies demonstrated that compound **2D216** acted as a potent co-adjuvant when used in combination with MPLA that enhanced antigen-specific IgG equivalent to that of AS01B. The combination adjuvant MPLA/**2D216** produced Th1 dominant immune responses and importantly protected mice from lethal influenza virus challenge. **2D216** alone or **2D216**/MPLA demonstrated minimal local reactogenicity and no systemic inflammatory response. In summary, **2D216** augmented the beneficial protective immune responses of MPLA as a co-adjuvant and showed an excellent safety profile.

## Introduction

Adjuvants are used to enhance the potencies of protein and peptide-based vaccines (1, 2). Common strategies in designing such adjuvants incorporate natural or semisynthetic components that stimulate the innate immune system. Ligands for pattern recognition receptors (PRRs) are critical activators of innate immunity. They include Toll-like receptors (TLRs) and C-type lectin receptors (CLRs), as well as the nucleotide-binding oligomerization domain (NOD)-like receptors (NLRs) and retinoic acid-inducible gene I (RIG-I) like receptors, which are being actively investigated for activity and safety as adjuvants (1). The FDA has approved a TLR4 ligand, monophosphoryl lipid A (MPLA), and a TLR9 ligand, CpG 1018, for clinical use as vaccine adjuvants (3–5). Despite these advances, single-agent adjuvants are not always sufficient to generate high titer protective antibody responses in vulnerable populations, including the elderly and immunocompromised hosts (3, 6, 7).

To address this medical need, we first examined published data on combinations of available adjuvants to augment protective immune responses. Preclinical data suggest that this is a promising avenue for the use of PRR ligands as adjuvants. The TLR4 adjuvant MPLA has been approved for use in co-adjuvant systems with AS04 (oil-in-water emulsion) and AS01B (saponin) (2, 8–10). Other co-adjuvant systems, including CAF01 with a CLR Mincle ligand and IC31 with a TLR9 ligand, ODN1, are currently being tested in clinical trials (11, 12).

The stimulation of PRRs commonly activates the NF-κB pathway, which plays a critical role in initiating signaling pathways that lead to the differentiation, maturation, and recruitment of antigen-presenting cells (APCs) (13). Thus, we postulated that small synthetic molecules that enhance NF-κB activation initiated by a TLR4 ligand could improve adjuvant efficacy. To obtain such molecules, we previously performed a cell-based high-throughput screening (HTS) campaign using the human monocytic cell line THP-1 equipped with an NF-κB fluorescence resonance energy transfer (FRET) reporter construct (14). Cells were simultaneously stimulated with lipopolysaccharide (LPS) as a TLR4 ligand and individual compounds from a small molecule compound library (~168K compounds) (14). A hit compound *N*-(4-(2,5-dimethylphenyl)thiazol-2-yl)-4-(piperidin-1-ylsulfonyl)benzamide (hereafter designated as compound **2D216**) was identified that prevented decay of NF-κB activation by LPS at 16 h incubation, without increasing the peak NF-κB activation at 5 h incubation (14). This characteristic attribute of sustaining LPS-induced NF-κB activation without further increasing early peak NF-κB activity suggested that such compound may be a safe and an effective co-adjuvant for MPLA, an approved TLR4 adjuvant.

In this report, we examined the adjuvant activity of **2D216** alone and in combination with MPLA *in vitro* and *in vivo*. As a single agent, the compound showed minimal activity on APCs including dendritic cells and B cells and no direct activity on T cells. However, when combined with a TLR4 ligand, **2D216** enhanced the transcription of chemokines, cytokines, and surface molecules associated with antigen presenting function. In the preclinical immunization models, the combination adjuvant (MPLA/**2D216**) augmented antigen-specific IgG levels equivalently to that of AS01B and induced a minimal local intramuscular infiltration of inflammatory cells and systemic inflammatory response.

## Materials and Methods

### Cells and Reagents

Mouse bone marrow-derived dendritic cells (mBMDCs) were generated from the femurs and tibias of mice as previously described (15). Mouse CD4^+^ T cells and CD19^+^ B cells were isolated from splenocytes using EasySep Mouse CD4 T Cell Isolation Kit (ST-19852, STEMCELL Technologies, Vancouver, Canada) and mouse CD19 MicroBeads (130-121-301, Miltenyi Biotec, Bergisch Gladbach, Germany) according to the manufacturer’s protocol. Cells were cultured in RPMI supplemented with 10% FBS (Omega, Tarzana, CA, USA) and penicillin/streptomycin (100 unit/mL/100 μg/mL, Thermo Fisher Scientific, Waltham, MA). Compound **2D216** was synthesized in our laboratory, and purity was confirmed by LC/MS. Ovalbumin (OVA) was purchased from Worthington Biochemical (Lakewood, NJ, USA). LPS (LPS-EB Ultrapure tlrl-eblps) and MPLA (vac-mpla) were purchased from InvivoGen (San Diego, CA, United States). AS01B was purchased from GlaxoSmithKline (GSK, Middlesex, United Kingdom, Zoster Vaccine Recombinant, Adjuvanted SHINGRIX).

### Animals

Six to eight-week-old BALB/c, C57BL/6, and DO11.10 mice were purchased from The Jackson Laboratory (Bar Harbor, ME, United States). *Myd88^-/-^* mice were a gift from Dr. Shizuo Akira (Osaka University, Osaka, Japan). *Ticam1^Lps2^* mice were kindly provided by Dr. Bruce Beutler (University of Texas Southwestern Medical Center, Dallas, TX, USA). All animal experiments received prior approval by the UC San Diego Institutional Animal Care and Use Committee (IACUC). The influenza challenge study was performed by the Institute for Antiviral Research of Utah State University. The study was approved by IACUC of Utah State University.

### nCounter Gene Expression Assay and Data Analysis

nCounter Mouse Immunology Panels (XT-CSO-MIM1-12, NanoString, Seattle, WA, United States), including a total of 561 genes, were used according to the manufacturer’s instruction. Briefly, mBMDCs (0.5×10^6^ cells/mL) were treated with 0.5% dimethylsulfoxide (DMSO) as vehicle, **2D216** (5 μM), LPS (1 ng/mL), or LPS/**2D216** for 5 h and total RNA was isolated. Data were acquired in triplicates, and the expression values were normalized using positive controls to eliminate platform related variation, negative controls to eliminate background effect, and housekeeping genes to remove variation due to sample input. Genes with zero counts were removed from group comparisons, linear models for microarray with the trend method were used to compare log2 expression values among groups. The Benjamini-Hochberg procedure was applied to control the false discovery rate (FDR). A gene was considered significantly changed if FDR < 0.05. Principal component analysis (PCA) was performed and plotted using Prism 9 software (GraphPad Software, San Diego, CA, United States) to assess similarity or dissimilarity between the datasets. Data are deposited and available through GEO with accession number GSE181134 (https://www.ncbi.nlm.nih.gov/geo/query/acc.cgi?acc=GSE181134:;!!LLK065n_VXAQ!yy4cAlugEz2j7waYDkyjNnKVpmEpSpvNg6DspkiEjVpYOpT_OMuq6sh4RWboK6I2wg$).

### Cytokine ELISA

Wild type, *Myd88^-/-^* and *Ticam^Lps2^* mBMDCs (0.5×10^6^ cells/mL), mouse CD19^+^ B cells (1×10^6^ cells/mL), and mouse CD4^+^ T cells (1×10^6^ cells/mL) were treated as detailed in each figure legend, and the culture supernatants were tested for IL-12 p40/p70, TNF-α, IL-6, and IL-2 by ELISA. Antibodies and reagents are listed in **Supplementary Table 1**.

### Antigen-Specific T Cell Proliferation Assay

mBMDCs (5×10^5^ cells/mL) from BALB/c mice were treated with **2D216** (5 μM) with or without MPLA (100 ng/mL) overnight and OVA protein (10 μg/mL) were loaded for 4 h. CD4^+^ T cells were isolated from spleens of DO11.10 T cell receptor (TCR) transgenic mice using EasySep Mouse CD4 T Cell Isolation Kit (ST-19852, STEMCELL Technologies) and labeled with 5-(and -6)-carboxyfluorescein diacetate succinimidyl ester (CFSE, 4 μM, C34554, Molecular Probe, Eugene, OR, United States). mBMDCs were washed twice and co-cultured with equal numbers of CFSE-labelled CD4^+^ T cells (5×10^5^ cells/mL) for 3 days. Supernatants were removed and stored at -20°C and later tested for IL-2 by ELISA. The T cells were stained with AF647-conjugated anti-DO11.10 TCR antibodies (BD Biosciences, United States) and analyzed with a MACSQuant Analyzer 10 (Miltenyi Biotec) for CSFE intensity in the TCR^+^ gated population. The % divided, the percent of the live CFSE-labelled gated T cells that entered division was calculated using FlowJo (version 10.6.1, Becton Dickinson, Ashland, OR, United States). Antibodies and reagents are listed in **Supplementary Table 1**. For direct activation of mouse primary CD4^+^ T cells, CD4^+^ T cells were treated with plate-bound anti-mouse CD3 (0.5 μg/mL, 16-0032-82 Thermo Fischer Scientific, Waltham, MA, United States) and soluble anti-mouse CD28 (1 μg/mL, Thermo Fischer Scientific, #16-0281-82) antibodies for 3 days.

### Analysis of CD40 and CD86 Expression

For co-stimulatory molecules and cell surface activation makers, mBMDCs and CD19^+^ B cells were treated for 24 h as described in each figure legend. Antibodies and reagents are listed in **Supplementary Table 1**.

### Immunoblot

mBMDC cells (5×10^5^ cells/mL) cells were rested overnight and then treated with 5 µM **2D216** in the presence or absence of 1 ng/mL LPS for indicated time periods. Treated cells were lysed with PhosphoSafe Extraction Reagent (71296, EMD Millipore, Billerica, MA, United States) mixed with protease inhibitor cocktail (11697498001, Roche, Manheim, Germany) and 0.1% SDS. Protein concentrations were determined using Pierce BCA protein assay kit (23225, Thermo Fisher Scientific). The reduced and denatured cell lysates (10 µg protein) were separated by gel electrophoresis and transferred onto PVDF membranes. The membranes were blocked with 5% bovine serum albumin (BSA) in 0.1% Tween-20 Tris-buffered saline (TBST). The membranes were incubated in primary antibody and secondary antibody diluted in 5% - BSA- TBST and signals were visualized with SuperSignal™ West Dura Extended Duration Substrate (34075, Thermo Fisher Scientific). Anti-phospho NF-*κ*B p65, and anti-phospho IRF3 (Ser396) and anti-actin were purchased from Cell Signaling Technology (Danvers, MA).

### Formulation of Antigen and Adjuvant for *In Vivo* Studies

The adjuvant and antigen solutions for immunization were separately prepared and then mixed together. For the combined adjuvant 50µl **2D216** (40 mM stock solution in DMSO) was added to 5µL MPLA (20 µg/mL stock solution). Separately, antigen OVA was prepared by adding 4 µL of OVA (50 mg/mL stock solution in saline) to a final volume of 445 µL saline. OVA in saline was then added to the adjuvant in DMSO solution prepared above and the mixture was vortexed to obtain 0.5 mL of vaccine formulation for injection (10 doses). To verify the homogeneity and stability of the vaccine formulation, we analyzed the sample by optical imaging. A freshly prepared formulation (0.5 mL) was added to a 1mL quartz cuvette and filmed for 30 minutes using a high-resolution smartphone camera (Samsung Galaxy S10, Korea) mounted at a fixed distance. Frames captured at 0, 5, 10, 15, 20, 25, and 30 minutes were extracted using Adobe Premier Pro CS5 software and three regions of interest (ROIs) were demarcated using ImageJ (Ver 1.53e) and the mean pixel density was calculated for each ROI (**Supplementary Figures 1A–D**). The formulation of OVA with MPLA and **2D216** retained its mean density and homogeneity for 30 minutes unlike the positive control compound which demonstrated aggregation and increased density in the bottom ROI with gravity over time (**Supplementary Figures 1B–D**).

### *In Vivo* Adjuvant Activity Study

BALB/c male and female mice (n = 5-10/group) were immunized in the right gastrocnemius muscle with model antigen ovalbumin (20 μg/mouse, Worthington Biochemicals, Lakewood, NJ, United States) mixed with **2D216** (200 nmol/mouse) and/or MPLA (10 ng/mouse) in a total volume of 50 μL on day 0 and day 21. 10% DMSO and AS01B (MPLA 1 μg and saponin QS-21 1 μg/mouse) were used as a vehicle and positive controls, respectively. On days 28 and 42, mice were bled, and OVA-specific IgG2a and IgG1 titers were measured by ELISA as previously described (16). For splenocyte re-stimulation analysis, spleens were harvested on day 28, and single-cell suspensions were prepared using MACS (Miltenyi Biotec). After red cell lysis using ammonium-chloride-potassium (ACK) lysing buffer (A1049201, Gibco, Waltham, MA, United States), splenocytes (5×10^6^ cells/mL) were re-stimulated with 100 μg/mL OVA protein for 3 days. IFN-γ in supernatants were measured using mouse IFN-γ ELISA kit (R&D Systems, Minneapolis, MN, United States). For germinal center reaction analysis, draining lymph nodes, including popliteal and inguinal lymph nodes, were harvested on day 28, and single-cell suspensions were stained with fluorescent antibodies (**Supplementary Table 1**) subjected to flow cytometry analysis.

### Maximal Feasible Dose Study

BALB/c female mice (n = 5/group) were intraperitoneally injected with 50 μ;g (110 nmol)/g mouse or 100 μg (220 nmol)/g mouse and body weights and behaviors were monitored for 7 days. Compound **2D216** was incorporated into a liposome formulation containing **2D216**, 1,2-dioleoyl-3-phosphatidylcholine (DOPC, Avanti polar Lipids, St. Louis, MO, United States) and cholesterol (C8667, Sigma) with a mol % ratio of 10:80:10, respectively, using the thin-film rehydration method in our laboratory to achieve high concentrations (17).

### Systemic and Local Reactogenicity Studies

BALB/c female mice (n = 3-5/group) were injected in the right gastrocnemius muscle with **2D216** (200 nmol/mouse) with or without MPLA (10 ng/mouse). AS01B was used as a control. After 2 and 24 h, mice were bled and sera were assessed for cytokine (TNF-α and IL-6) and C-reactive protein (CRP) levels by Luminex multiplex cytokine assay (Thermo Fischer Scientific) and ELISA. After 24 h, the right gastrocnemius muscles were harvested and histological sections with 5 μm thickness were stained with hematoxylin and eosin (H&E) and analyzed by BZ-X810 microscopy (KEYENCE, Osaka, Japan).

### Influenza Challenge Test

The influenza challenge test was performed at the Institute for Antiviral Research of Utah State University as previously described (17). Briefly, BALB/c mice (n = 10/group) were intramuscularly immunized with **2D216** (100 nmol/mouse) with or without MPLA (1 μg/mouse) as an adjuvant and inactivated influenza A virus [IIAV, A/California/07/2009, (H1N1)pdm09] (0.3 μg/mouse) as an antigen in a total volume of 50 µL on day 0 and intranasally challenged with approximately 1×10^5^ (3×LD_50_) cell culture infectious doses (CCID_50_) of homologous influenza A virus, (H1N1)pdm09 in 50 µL on day 21. Body weights and survival of mice were monitored. Virus neutralization and hemagglutination-inhibition (HAI) titers were determined by Antiviral Research of Utah State University as previously described (17).

### Statistical Analysis

Data obtained *in vitro* studies are shown as means with standard deviation (SD), and *in vivo* data are presented as means with standard error of the mean (SEM). For *in vitro* data, Mann-Whitney *U* test to compare two groups was used. For multiple comparisons of *in vivo* data, one-way ANOVA with Dunnett’s *post hoc* test or Kruskal-Wallis tests with Dunn’s *post hoc* test was applied. For body weights, a last-value-carried-forward approach was used to impute missing values after a mouse was sacrificed, the average weight over time was used as an outcome for comparison, and Kruskal-Wallis tests with Dunn’s *post hoc* test was applied. A log-rank test was used to test for a significant difference between Kaplan-Meier survival curves. Prism 9 software (GraphPad Software) was used. A p-value less than 0.05 was considered statistically significant.

## Results

### 2D216 Augments TLR4 Stimulation of Murine Bone Marrow-Derived Dendritic Cells

**Figure 1:**
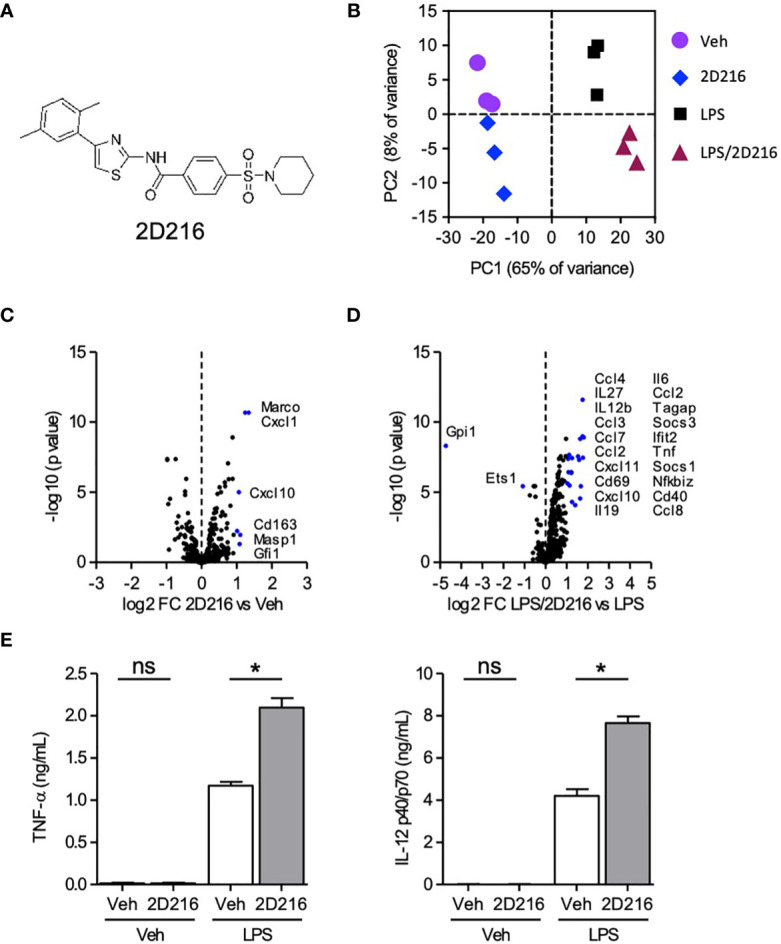
Augmentation of TLR4 ligand-mediated activation of mBMDCs by **2D216**. **(A)** Chemical structure of compound **2D216**. **(B–D)** nCounter gene expression analysis of genes upregulated by LPS/**2D216** and LPS. mBMDCs (1×10^6^ cells/mL) were incubated with vehicle (Veh), **2D216** (5 μM), LPS (1 ng/mL), or **2D216** (5 μM) plus LPS (1 ng/mL) for 5 h, and total RNAs were subjected to nanoString nCounter^®^ Mouse Immunology Panel analysis containing 561 mouse immune-related genes. **(B)** PCA demonstrates the clustering of the treatments. **(C)** Genes significantly up-regulated by **2D216** compared to Veh and **(D)** LPS/**2D216** compared with LPS alone are shown as blue dots (log2 FC > 1 and FDR < 0.05). **(E)** Cytokine secretion induced by **2D216**. mBMDCs (0.5×10^6^ cells/mL) were incubated with Veh, **2D216** (5 μM), LPS (1 ng/mL), or LPS (1 ng/mL)/**2D216** (5 μM) plus LPS (1 ng/mL) for 20 h, and the levels of IL-12 p40/p70 and TNF-α in the LPS/**2D216** culture supernatants were significantly greater than LPS alone. Data represent mean ± SD of triplicates of two independent experiments showing similar results. *p < 0.05 by Mann-Whitney *U* test compared to Veh/Veh or MPLA/Veh. ns, not significant.

In the HTS campaign, we identified a hit compound *N*-(4-(2,5-dimethylphenyl)thiazol-2-yl)-4-(piperidin-1-ylsulfonyl)benzamide (**2D216,** **Figure 1A**), which prolonged NF-κB reporter activation induced by LPS, a TLR4 ligand, as a primary stimulus. Thus, we hypothesized that this compound might enhance the activation of APCs in combination with a TLR4 ligand (e.g., the FDA-approved adjuvant MPLA). To test this hypothesis, we resynthesized the compound and confirmed its purity (> 99%). To assess the innate immune activation profile of **2D216** in the presence and absence of TLR4 ligand, we examined mRNA transcript levels after stimulating primary mBMDCs by nCounter^®^ gene expression assay (NanoString). Principle component analysis (PCA) indicated that **2D216** has a limited intrinsic activity that was distinct from vehicle (Veh) and LPS, and the combination of LPS/**2D216** was clustered separately from LPS alone (**Figure 1B**). **2D216** alone upregulated expression (2 fold) of only six transcripts compared to Veh stimulated cells (**Figure 1C**). In contrast, the skewing volcano plots between LPS and LPS/**2D216** demonstrated that expression levels of 22 genes were enhanced upon LPS/**2D216** treatment (**Figure 1D**). These molecules included cell surface activation markers as well as chemokines and cytokines. We selected two analytes and confirmed that **2D216** significantly enhanced secretion of IL-12 and TNF-α by LPS co-stimulated mBMDCs, while **2D216** alone was insufficient for detectable cytokine release (**Figure 1E** and **Supplementary Figures 2A–C**).

TLRs signal through two major intermediaries: Myeloid differentiation primary response 88 (MyD88) and the TIR domain-containing adapter-inducing interferon-β (TRIF) protein. TLR4 is the only TLR that utilizes both pathways and TLR7 only utilizes MyD88 (13). We tested the ability of **2D216** to augment the cytokine production induced by LPS and a TLR7 ligand, **1V270** (16), in cells deficient in MyD88 and TRIF (**Supplementary Figures 2A–C**). **2D216** treatment enhanced the cytokine production in wild type cells for both ligands. There was no TNF or IL-12 production induced in the MyD88 cells, however the level of RANTES induced by LPS (through the TRIF pathway) was greater in the **2D216** treated cells. In the *Ticam^Lps2^* (TRIF mutant) cells the **2D216** and **1V270** cotreated cells produced significantly higher TNF, and IL-12 than cells treated with **1V270** alone. These results indicate that **2D216** can enhance cytokine production for another TLR ligand and is not exclusive to TLR4. **2D216** does not stimulate phosphorylation of NF-κB p65 or interferon regulatory factor (IRF)3 (**Supplementary Figure 2D** and **Supplementary Figures 3A–C**) which further demonstrated that **2D216** is not active directly targeting MyD88, TRIF or their major downstream signaling pathways.

### 2D216 Enhances Activation of Antigen Presenting Cells and Does Not Directly Augment T Cell Activation *In Vitro*

**Figure 2:**
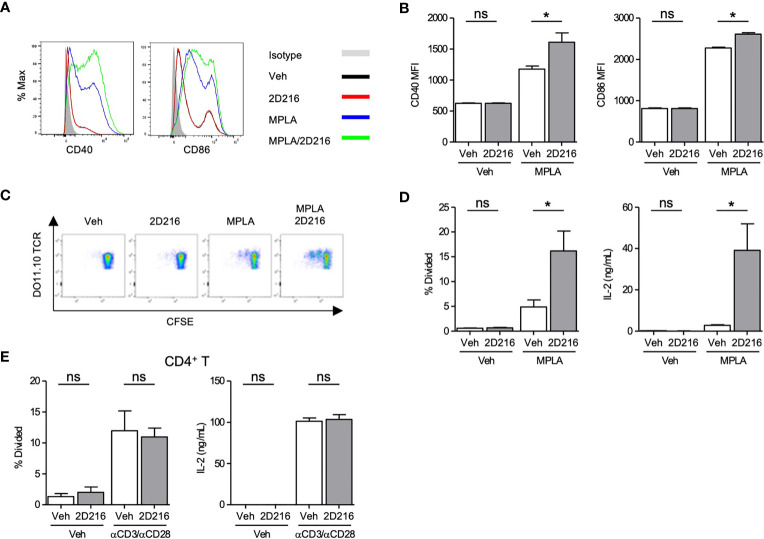
**2D216** enhances MPLA-induced antigen presenting function in mBMDCs. **(A, B)** Co-stimulatory molecule expression induced by **2D216**. mBMDCs (1×10^6^ cells/mL) were incubated with vehicle (Veh), **2D216** (5 μM), MPLA (100 ng/mL), or **2D216** (5 μM) plus MPLA (100 ng/mL) for 24 h and expression of CD40 and CD86 were compared by mean fluorescent intensity (MFI) analyzed using flow cytometry, *p < 0.05 by Mann-Whitney *U* test compared to Veh/Veh or MPLA/Veh. **(C)** Representative plots of T cell proliferation with MPLA/**2D216** treated mBMDCs in co-culture. mBMDCs (0.5×10^6^ cells/mL) were treated with Veh, **2D216** (5 μM), MPLA (100 ng/mL), or MPLA (100 ng/mL)/**2D216** (5 μM) overnight, and pulsed with OVA (10 μg/mL) for 4 h. Antigen-pulsed mBMDCs were then co-cultured with the same number of CFSE-labelled CD4^+^ DO11.10 T cells for 3 days. **(D)** T cell proliferation was determined by CFSE dilution. Percent T cells that divided relative to the original population is shown. IL-2 released in the culture supernatant was determined by ELISA. **(E)** Primary mouse splenic CD4^+^ T cells were isolated, CFSE-labeled and stimulated with plate-bound anti-CD3 antibodies (0.5 μg/mL) and soluble anti-CD28 antibodies (1 μg/mL) with or without **2D216** (5 μM) for 3 days. T cell proliferation was determined by CFSE dilution. IL-2 levels in the culture supernatant were measured by ELISA. Data represent mean ± SD of triplicates of two independent experiments showing similar results. *p < 0.05 by Mann-Whitney *U* test compared to Veh/Veh or MPLA/Veh. ns, not significant.

**2D216** had minimal intrinsic effects on mBMDC gene expression and cytokine production without a primary stimulus (**Figure 1**). **2D216,** when used in conjunction with a TLR4 ligand, significantly upregulated surface markers for APC activation, CD40 and CD86, compared to either **2D216** or TLR4 ligand alone (**Figures 2A, B**). **2D216** also enhanced LPS-induced expression of activation markers (CD86 and CD69) and secretion of IL-6 in primary mouse splenic CD19^+^ B cells *in vitro* (**Supplementary Figures 4A–C**). To assess if **2D216** would be effective in enhancing antigen-presenting function and cognate T cell priming, mBMDCs stimulated by **2D216/**MPLA were co-cultured in the presence of antigen with CFSE-labeled CD4^+^ T cells expressing OVA-specific T cell receptors (from DO11.10 mice) (**Figures 2C, D**). mBMDCs treated with OVA and MPLA/**2D216** induced significantly higher T cell proliferation and IL-2 release than those by treatment of MPLA alone (**Figures 2C, D**). To examine the possibility that **2D216** directly activates T cells to enhance their proliferation, anti-CD3/anti-CD28 antibody-activated primary splenic CD4^+^ T cells were incubated with **2D216** (**Figure 2E**). **2D216** had no effect on proliferation and IL-2 release by T cells in the presence and absence of TCR engagements (**Figure 2E**). These results suggested that **2D216** likely augmented the APC activation by MPLA in the co-culture system with minimal effects on T cells.

### 2D216 Enhances Th1-Biased Adjuvant Activity *In Vivo*

**Figure 3:**
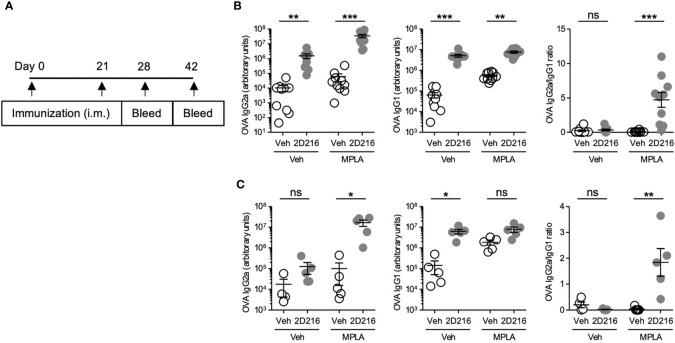
**2D216** enhances MPLA-induced Th1-dominant adaptive immune responses in female mice. **(A)** Female BALB/c mice (n = 5-10/group) were i.m. immunized with OVA (20 μg/mouse) adjuvanted with vehicle (Veh; 10%DMSO in saline), **2D216** (200 nmol/mouse), MPLA (10 ng/mouse), or a combination (MPLA/**2D216)** and bled on day 28 and day 42. OVA-specific IgG2a and IgG1 levels in sera from day 28 **(B)** and day 42 **(C)** were assayed by ELISA. IgG2a/IgG1 ratio was calculated. Data shown are means ± SEM of two independent experiments showing similar results. *p < 0.05, **p < 0.01, ***p < 0.001 by Kruskal-Wallis tests with Dunn’s *post hoc* test. ns, not significant.

The above results demonstrated that **2D216** acted as a co-adjuvant with TLR4 ligands in mBMDCs and B cells *in vitro*. In vivo co-adjuvant activity of **2D216** was examined in male and female mice separately. Female BALB/c mice were intramuscularly (i.m.) immunized on days 0 and 21 with the model antigen OVA adjuvanted with **2D216** alone or in combination with MPLA (MPLA/**2D216**) (**Figure 3A**). Inoculation with **2D216** as a single adjuvant significantly enhanced the levels of anti-OVA IgG2a and IgG1 produced; however, a greater increase was seen in mice immunized with MPLA/**2D216**-adjuvanted OVA at both 28 and 42 days (**Figures 3B, C**). The levels of anti-OVA IgG2a demonstrated a decline at 42 days when **2D216** was used as a single adjuvant; however, anti-OVA IgG2a levels were sustained when used in combination with MPLA (**Figure 3C**).

**Figure 4:**
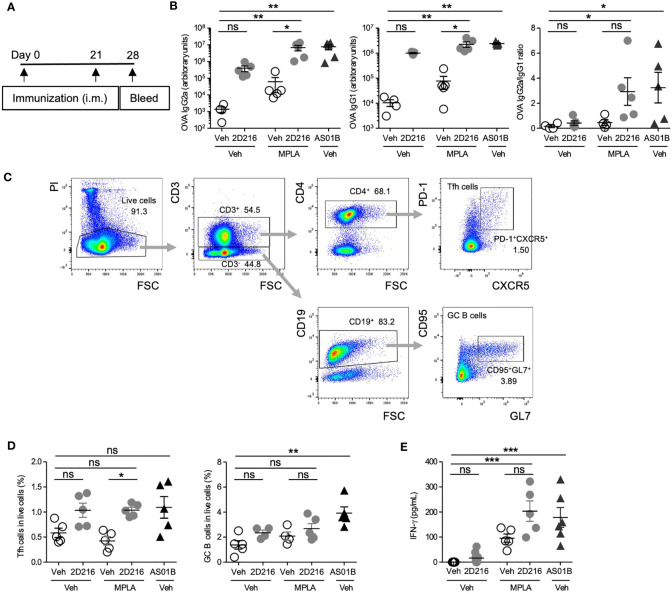
MPLA/**2D216** induced Th1-dominant cellular immune responses in male mice. **(A)** Male BALB/c mice (n = 5/group) were i.m. immunized with OVA protein (20 μg/mouse) adjuvanted with vehicle (Veh;10%DMSO in saline), **2D216** (200 nmol/mouse), MPLA (10 ng/mouse), or MPLA/**2D216,** and bled on day 28. **(B)** OVA-specific IgG2a and IgG1 levels in sera on day 24 were assayed by ELISA. IgG2a/IgG1 ratios were calculated. **(C, D)** On day 28, draining lymph nodes were harvested, and Tfh and GC B cells were examined by flow cytometry analysis. The gating strategy is shown **(C)**. Percent Tfh cells (CD3^+^CD4^+^PD-1^+^CXCR5^+^) and GC B cells (CD3^-^CD19^+^CD95^+^GL7^+^) in the gated live cells are shown **(D)**. **(E)** On day 28, splenocytes (5×10^6^ cells/mL) were harvested and re-stimulated with OVA (100 μg/mL) for 3 days. The levels of IFN-γ in the culture supernatants were measured by ELISA. Data shown are means ± SEM of two independent experiments showing similar results. *p < 0.05, **p < 0.01, ***p < 0.001 by Kruskal-Wallis tests with Dunn’s *post hoc* test. ns, not significant.

In a separate cohort, male BALB/c mice were immunized, i.m. on days 0 and 21, with OVA adjuvanted with **2D216** alone or in combination with MPLA (**Figure 4A**). An additional group with FDA-approved combination adjuvant AS01B (MPLA/saponin) was added as a comparator. On day 28, **2D216** enhanced MPLA-induced OVA-specific IgG2a by over 100 fold, while antigen-specific IgG1 production showed lesser increase in mice vaccinated with **2D216** alone compared to vehicle adjuvanted OVA (**Figure 4B**). The levels of anti-OVA antibodies were equivalent between mice immunized with the two combination adjuvants (AS01B as MPLA/saponin or MPLA/**2D216**) (**Figure 4B**). When IgG2a: IgG1 ratios were plotted to evaluate Th1/Th2 balance, **2D216** significantly enhanced Th1-biased immunoglobulin responses when combined with MPLA (**Figure 4B**). On day 28, the draining lymph nodes were removed and analyzed for follicular helper T cells (Tfh) and germinal center (GC) B cells, both of which are important for sustained humoral responses in draining lymph nodes (18) (**Figures 4C, D**). Immunization with **2D216** in combination with MPLA increased the proportions of Tfh cells compared to MPLA/Veh (**Figure 4D**, left panel), while **2D216** showed minimal effects on the GC B cell population (**Figure 4D**, right panel). When splenocytes harvested on day 28 were restimulated *ex-vivo* with OVA for 3 days, cells from mice immunized with the combination adjuvant, MPLA/**2D216** and AS01B, secreted significantly higher levels of IFN-γ into the culture supernatants compared to the vehicle control (**Figure 4E**). These data further support that **2D216** augments Th1 responses induced by MPLA.

### 2D216 Shows Minimal Local and Systemic Reactogenicity

**Figure 5:**
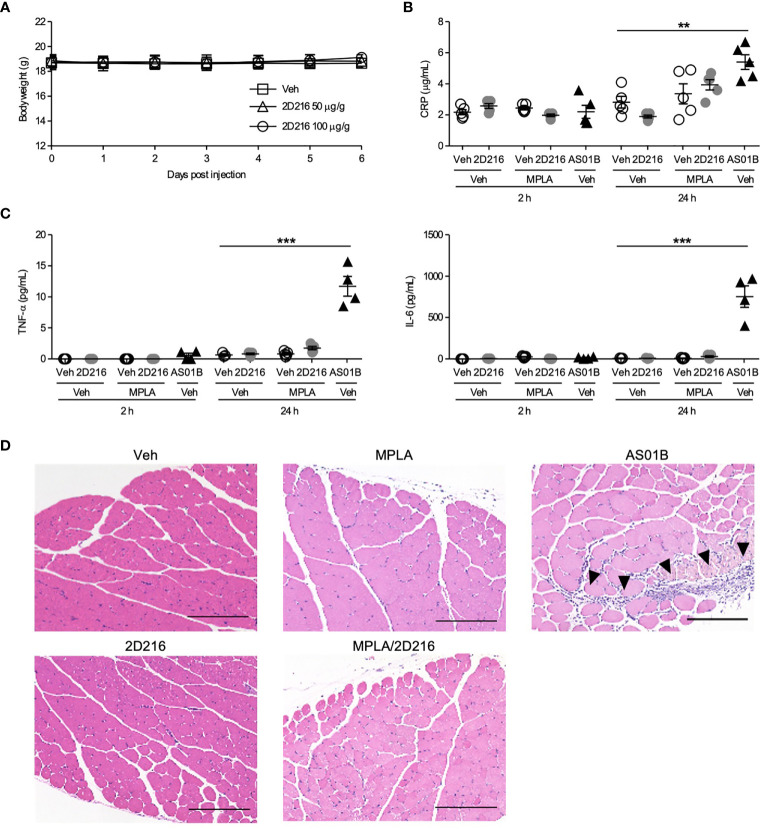
MPLA/**2D216** shows a minimal local and systemic reactogenicity. **(A)** Maximal feasible dose test. BALB/c mice (n = 5/group) were intraperitoneally injected with vehicle (Veh; 10%DMSO in saline), or **2D216** (50 and 100 μg/g mouse) on day 0 and body weights were monitored for 7 days. **(B–D)** BALB/c mice (n = 3-5/group) were i.m. injected with Veh, **2D216** (200 nmol/mouse), MPLA (10 ng/mouse), MPLA/**2D216** or AS01B (10 μL/mouse). Two and 24 h after the injection, sera were collected, and CRP **(B)**, TNF-α **(C)**, and IL-6 **(C)** levels were determined by ELISA. Data shown are means ± SEM of two independent experiments showing similar results. **p < 0.01, ***p < 0.001 by one-way ANOVA with Dunnett’s *post hoc* test compared to Veh/Veh. **(D)** Local reactogenicity. After 24 h, the injected muscles were harvested. Examples of H&E stained histological sections are shown (scale bar 200 μm). Arrowheads indicate the areas with inflammatory cell infiltration. ns, not significant.

To address compound safety, a maximal feasible dose of **2D216** (100 μg/g mouse body weight) was intraperitoneally administered to the mice. The injection of highest dose of **2D216** had no effects on body weight and behavioral changes for 7 days (**Figure 5A**). To examine systemic and local reactogenicity of the combination adjuvant of MPLA/**2D216**, we i.m. immunized BALB/c mice with **2D216** alone or in combination with MPLA, and compared the effects to AS01B. Sera were collected at 2 and 24 h post-injection, and inflammatory markers, CRP, TNF-α and IL-6, were measured. **2D216** alone or a combination with MPLA did not result in an elevation of these inflammatory markers, while the use of AS01B significantly increased the levels of these inflammatory proteins in the sera of mice 24 h post-injection (**Figures 5B, C**). Muscle tissues at injection sites were harvested 24 h post-injection. The histologic examination showed that neither **2D216** alone nor in combination with MPLA resulted in excess inflammatory cell infiltration or muscle necrosis (**Figure 5D**). In contrast, inflammatory cell infiltration areas in the muscle tissues injected with AS01B were observed (**Figure 5D**). Collectively, these data indicate that the combination adjuvant of **2D216** and MPLA has an acceptable safety profile.

### Combination Adjuvant With 2D216 Protects Mice From Lethal Influenza Challenge

**Figure 6:**
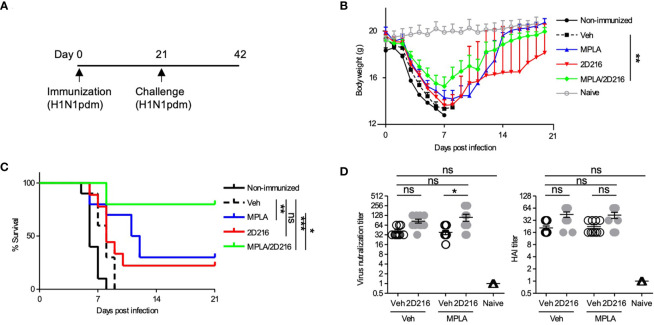
MPLA/**2D216** adjuvanted IIAV vaccination protects mice from lethal influenza virus challenge. **(A)** BALB/c mice (n = 10/group) were immunized with IIAV adjuvanted with vehicle (Veh; 10%DMSO in saline), MPLA (1 μg/mouse), **2D216** (100 nmol/mouse), or MPLA/**2D216** on day 0 and were infected with homologous influenza virus (H1N1)pdm09 on day 21. Body weights and survival were monitored for 21 days. **(B)** Body weights are indicated as mean ± SEM. For statistical analysis, last-value-carried-forward approach was used to impute missing values after a mouse died. The average weight over time was used as an outcome for comparison. *p < 0.05, **p < 0.01, ***p < 0.001 by Kruskal-Wallis tests with Dunn’s *post hoc* test. **(C)** Survival rates of mice post-challenge with homologous influenza virus. Kaplan-Meier curves with the log-rank test are shown. *p < 0.1, **p < 0.01, ***p < 0.0001, and ns, not significant. **(D)** The sera were collected from the mice prior to lethal challenge and tested for titer against the HA protein and viral neutralization. Data shown are means ± SEM. *p < 0.05, **p < 0.01, ***p < 0.001 by Kruskal-Wallis tests with Dunn’s *post hoc* test.

The adjuvant effects of MPLA/**2D216** were further examined in a lethal influenza virus challenge model in mice. BALB/c mice were i.m. immunized once with inactivated influenza A virus (IIAV) [A/California/07/2009, (H1N1)pdmCal9] adjuvanted with Veh, MPLA, **2D216**, and MPLA/**2D216** on day 0 and were intranasally challenged with homologous influenza virus (H1N1)pdm09 on day 21 (**Figure 6A**). The combination of MPLA/**2D216** significantly suppressed body weight loss after the virus challenge (**Figure 6B**). While MPLA improved the survival of mice from the viral challenge compared to the mice immunized with vehicle adjuvanted IIAV, **2D216** in combination with MPLA had the greatest potency in protecting the mice from the lethal virus challenge (**Figure 6C**). The viral neutralization titers from sera in the mice immunized with MPLA/**2D216** adjuvanted IIAV were greater than those of the MPLA/Veh group, while **2D216** showed minimal effects on hemagglutinin inhibition titers (HAI) (**Figure 6D**). These results support that the combination of MPLA and **2D216** is a promising candidate as an effective co-adjuvant system.

## Discussion

Combination vaccines such as AS01B, including a TLR4 ligand, MPLA, have shown improved humoral and cellular responses and overcome reduced immune responses in the elderly for shingles and influenza (4, 7). However, undesired local and systemic reactogenicity can reduce compliance, especially if a second vaccination is required (4, 19, 20). To overcome this problem, we conducted HTS to identify non-toxic synthetic adjuvants that enhanced TLR4 ligand-mediated immune responses (14). Here we have described one of the compounds, **2D216,** that enhanced immune responses triggered by TLR4 ligand MPLA in a cell type-specific manner. The combination of MPLA and **2D216** demonstrated Th1-biased humoral and cellular immune responses with less local and systemic reactogenicity and efficient protection of mice from lethal influenza challenge, suggesting that the combination adjuvant MPLA/**2D216** will be a promising potent vaccine adjuvant.

Although this study demonstrated that **2D216** could augment cellular activation by TLR ligands we did not observe a dependence of the compound on the MyD88 or TRIF signaling pathways. The direct target is likely to be upstream directly proximal to or on the cell membrane. However, our studies revealed the interesting cell-type-specific action of **2D216**. Concordantly, **2D216** enhanced LPS-induced activation and cytokine secretion in primary mouse CD19^+^ B cells, but not anti-CD3/anti-CD28 antibody-induced proliferation and cytokine secretion in primary mouse CD4^+^ T cells. Direct activation of B cells by vaccine adjuvants provides beneficial effects on host defense. For example, engagement of TLR7, C3d, and BAFF receptors on B cells by their ligands enhances antibody titer against influenza virus (21, 22). On the contrary, direct activation of T cells might lead to severe adverse effects such as cytokine storm (23–25). Hence, the activation of myeloid and B cells, but not T cells by **2D216,** is desirable for safe vaccine adjuvants.

The OVA immunization experiment demonstrated that a combination vaccine of MPLA and **2D216** induced Th1-biased humoral and cellular immune responses. This finding was consistent with the *in vitro* observation that **2D216** enhanced MPLA-induced mBMDC activation such as IL-12 secretion. Inducing Th1-biased T cell responses, especially in the immune senescent elderly, is important for protection against influenza infection (26, 27). This can explain the superior protection by MPLA/**2D216** combination compared to single treatment of each compound in mice against influenza challenge study. Although **2D216** alone had a minor effect on mBMDCs *in vitro*, it induced robust IgG1 titer *in vivo*. As reported for Alum and MF95 *in vivo*, some adjuvants induce primarily Th2 responses *via* multiple pathways, including depot effects, inflammasome activation, and effects on non-immune cells such as muscle cells (28–31). These pathways may contribute to Th2 responses elicited by **2D216** alone. Although these early studies are promising the addition of compound directly to MPLA in saline may have led to some inhomogeneity and microaggregation and future studies to improve the formulation may further enhance protective antibody responses.

In conclusion, we have identified and characterized the mode of action and *in vivo* adjuvanticity and safety of the new synthetic adjuvant **2D216**. **2D216** enhanced innate immune responses induced by TLR4 ligand, MPLA. This co-adjuvant effect of **2D216** is cell type-specific, observed in myeloid and B cells but not T cells. In OVA immunization and influenza challenge studies, combination adjuvant, MPLA/**2D216,** induced Th1-biased humoral and cellular responses and protected mice from lethal viral challenge. MPLA/**2D216** caused no local and systemic reactogenicity as compared to AS01B. Thus, **2D216** may become a promising candidate for future development as a vaccine co-adjuvant.

## Data Availability Statement

The raw data supporting the conclusions of this article will be made available by the authors, without undue reservation.

## Ethics Statement

Animal studies were reviewed and approved by UCSD IACUC and Utah State University IACUC.

## Author Contributions

TS, DC, MCo, and TH designed the study. TS, YS, FS-K, SY, FL, THo and JS performed experiments. NS, MCh, PC, and HC synthesized the compound. TS, KM and MP performed statistical analyses. TS, DC, MC, and TH interpreted data and wrote the manuscript. All authors contributed to the article and approved the submitted version.

## Funding

This study was supported by the National Institute of Health/National Institute of Allergy and Infectious Diseases under contracts HHSN272201400051C and 75N93019C00042 (Principal Investigator-DC).

## Conflict of Interest

The authors declare that the research was conducted in the absence of any commercial or financial relationships that could be construed as a potential conflict of interest.

## Publisher’s Note

All claims expressed in this article are solely those of the authors and do not necessarily represent those of their affiliated organizations, or those of the publisher, the editors and the reviewers. Any product that may be evaluated in this article, or claim that may be made by its manufacturer, is not guaranteed or endorsed by the publisher.
