# Supplementary material for: Small Molecule Potentiator of Adjuvant Activity Enhancing Survival to Influenza Viral Challenge
Source: Front Immunol. 2021 Sep 28;12:701445. doi: 10.3389/fimmu.2021.701445 (PMC8505803; doi:10.3389/fimmu.2021.701445)
Supplement: Supplementary file 1 [file DataSheet_1.pdf]

Supplementary Material

Supplementary Table 1. Reagents used in ELISA, flow cytometry, and immunoblots

Reagents	Dilution factor	Source	Catalog #
**ELISA**			
Capture antibodies			
Purified rat anti-mouse TNF	200	BD Biosciences	559064
Purified rat anti-mouse IL-12	200	BD Biosciences	551219
Purified rat anti-mouse IL-6	100	BD Biosciences	554400
Monoclonal rat anti-mouse CRP	100	Novus biologicals	NAB1829
Detecting antibodies			
Biotin rat anti-mouse TNF	1000	BD Biosciences	554415
Biotin rat anti-mouse IL-12	1000	BD Biosciences	554476
Biotin rat anti-mouse IL-6	1000	BD Biosciences	554402
Biotin goat anti-mouse CRP	1000	BD Biosciences	BAF1829
IgG2a-AP goat anti-mouse	2000	Southern Biotech	1080-04
IgG1-AP goat anti-mouse	2000	Southern Biotech	1070-04
Other reagents			
Streptavidin, HRP	1000	Thermo FisherScientific	43-4323
KPL SureBlue^{TM} TMB Peroxidase Substrate		Seracare	5120-0077
p-Nitrophenyl Phosphate tablets (pNPP)		Sigma	N2770
**ELISA kit**			
Mouse IL-2 Duo Set		R&D systems	DY402
Mouse IFN-γ Duo Set		R&D systems	DY485
**Flow cytometry**			
PE anti-mouse CD40	200	eBioscience	12-0401
APC/Cy7 anti-mouse 86	200	BioLegend	105030
APC anti-mouse CD69	200	BioLegend	104514
AF647 anti-mouse DO11.10 TCR	300	BD Biosciences	562524
BV510 anti mouse CD3	200	BD Biosciences	563024
FITC anti-mouse CD19	500	BD Biosciences	553785
e450 anti-mouse CD4	1500	eBioscience	48-0042
PE/Cy7 anti-mouse CD95	500	BD Biosciences	557653
APC anti-mouse CD138	200	BD Biosciences	558626
Pacific Blue anti-mouse GL7	350	BioLegend	144614
APC anti-mouse PD-1	150	BD Biosciences	562671
Biotin anti-mouse CXCR5	50	BD Biosciences	551960
Streptavidin PE	500	BD Biosciences	554061
**Immunoblot**			
anti-phospho IRF3 (Ser396) rabbit	1000	Cell Signaling Technology	4947
anti-phospho NF-κB p65 rabbit	1000	Cell Signaling Technology	3033
β-Actin (8H10D10) Mouse mAb	10,000	Cell Signaling Technology	3700
Anti-mouse IgG, HRP-linked	5000	Cell Signaling Technology	7076
Anti-rabbit IgG, HRP-linked antibody	5000	Cell Signaling Technology	7074
