# Supplementary material for: Small Molecule Potentiator of Adjuvant Activity Enhancing Survival to Influenza Viral Challenge
Source: Front Immunol. 2021 Sep 28;12:701445. doi: 10.3389/fimmu.2021.701445 (PMC8505803; doi:10.3389/fimmu.2021.701445)
Supplement: Supplementary file 2 [file DataSheet_2.pdf]

Supplementary Material

Supplementary Material

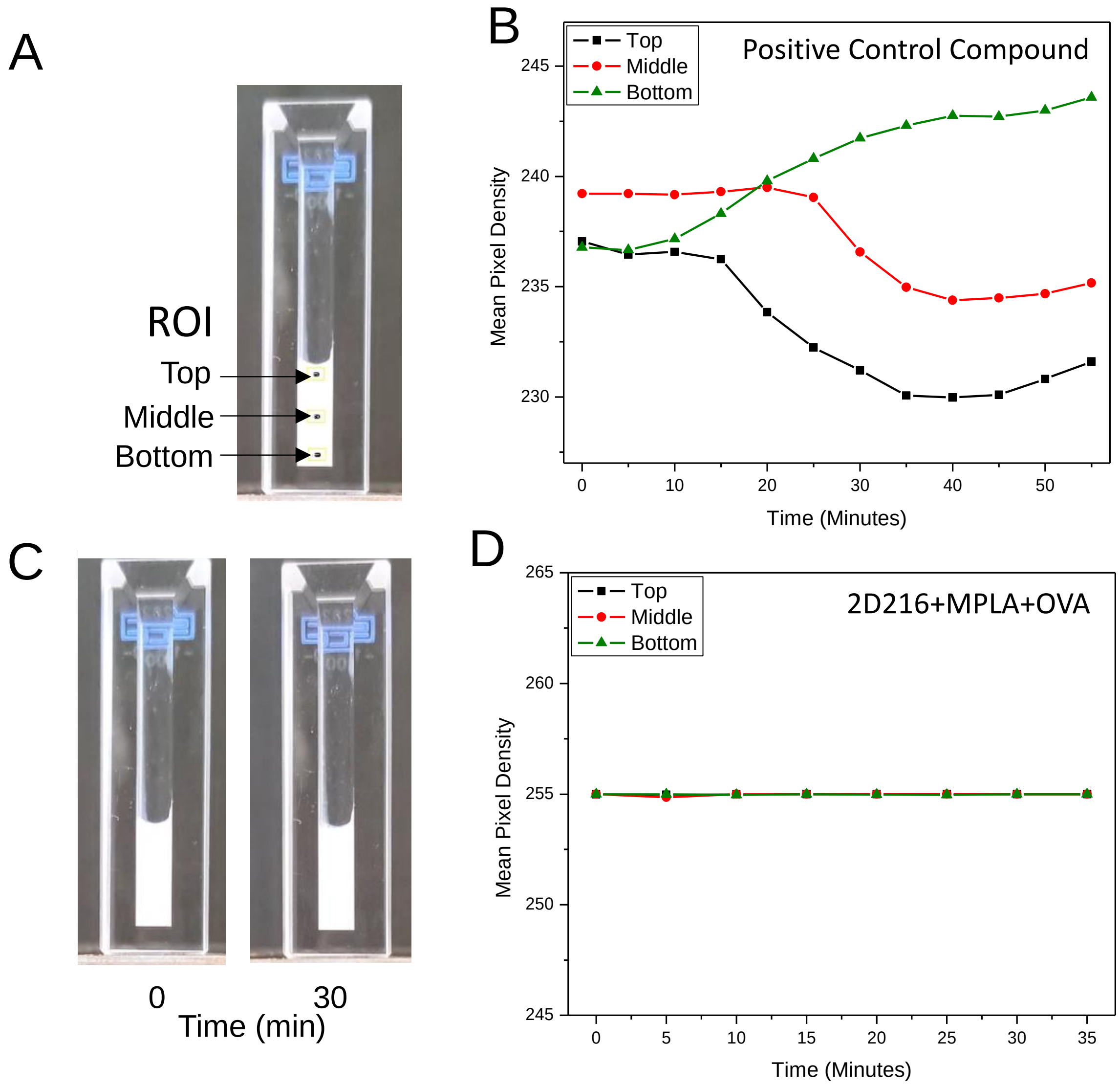

Supplemental Figure 1. Adjuvant formulation with 2D216, and MPLA does not aggregate over 30 minutes. (A) A quartz cuvette with the adjuvant admixture was videoed digitally and three regions of interest (ROI; top, middle, and bottom) were selected. (B) The mean pixel densities over time are plotted for a control compound known to aggregate. The pixel density of the bottom region increases as the densities of the middle and top regions decline as aggregates sediment with gravity. (C) Compound **2D216** (200 nmoles/dose) and MPLA (10 ng/dose) were mixed in DMSO/saline (10% v/v, total volume 0.5mL for 10 doses). (D) The adjuvant was videoed for settling changes during 35 minutes in a cuvette and mean pixel densities remained stable across all ROIs.

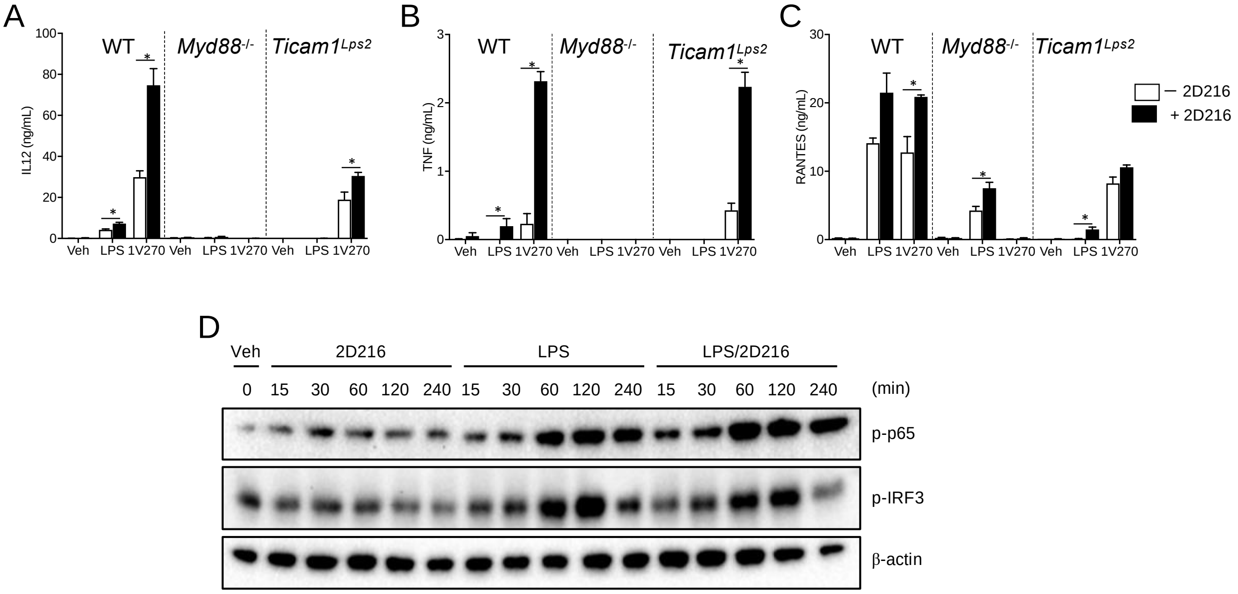

Supplemental Figure 2. 2D216 potentiates TLR activation in *$Myd88^{-/-}$* and *$Ticam^{Lps}$* mBMDCs. (**A-C**) **2D216** increases cytokine production in TLR ligand stimulated *$Myd88^{-/-}$* and *$Ticam^{Lps2}$* mBMDCs. mBMDCs (0.5×10^6 cells/mL) prepared from wild type (WT), *$Myd88^{-/-}$* and *$Ticam^{Lps2}$* mice were incubated with Veh, **2D216** (5 μM), LPS (1 ng/mL), **1V270** (TLR7 ligand, 1 μM), or the combination of **2D216** with LPS or **IV270** for 20 h, and the levels of IL-12 p40/p70, TNF-α, and RANTES in the culture supernatants were measured by ELISA. Data represent mean ± SD of triplicates of two independent experiments showing similar results. *$p < 0.05$ by Mann-Whitney *U* test. (**D**) 2D216 does not inherently stimulate NF-κB p65 and IRF3 phosphorylation. mBMDCs were treated with **2D216** (5 μM), LPS (1 ng/mL), or **2D216** (5 μM) plus LPS (1 ng/mL) up to 240 min, and the phosphorylation of NF-κB p65 and IRF3 were detected by immunoblot with phospho-specific antibodies against each protein. β-actin was used as a loading control. The original blots are shown in Supplemental Figure 2.

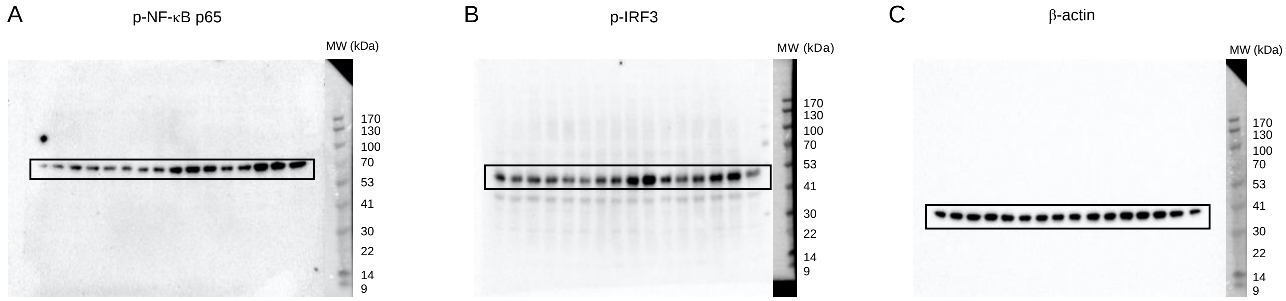

Supplemental Figure 3. Full original immunoblots presented in Supplemental Figure 2 for (A) p-NF-κB p65, (B) p-IRF3 and (C) β-actin.

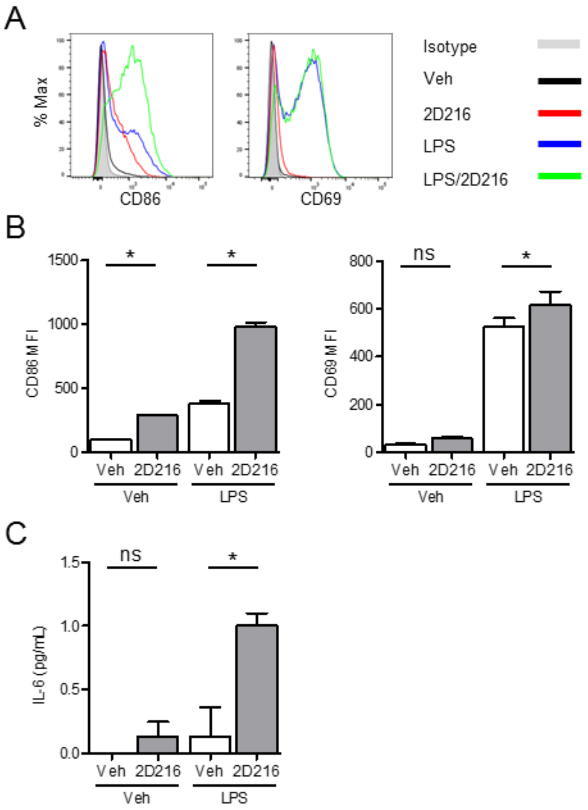

Supplemental Figure 4. Activation of primary CD19$^+$ B cells by 2D216. CD19$^+$ B cells were isolated from mouse splenocytes and treated with **2D216** (5 μM) in the presence or absence of LPS (100 ng/mL) for 24 h. The cells were stained with anti-CD69 and CD86 antibodies and analyzed by flow cytometry for activation markers CD69 and CD86 (A and B). IL-6 levels in the culture supernatant were determined by ELISA (C). Data represent mean ± SD of triplicates of two independent experiments showing similar results. *p < 0.05 by Mann-Whitney *U* test compared to Veh/Veh or MPLA/Veh.
